# The concomitant use of lapatinib and paracetamol - the risk of interaction

**DOI:** 10.1007/s10637-018-0573-1

**Published:** 2018-02-20

**Authors:** Agnieszka Karbownik, Edyta Szałek, Katarzyna Sobańska, Tomasz Grabowski, Agnieszka Klupczynska, Szymon Plewa, Anna Wolc, Magdalena Magiera, Joanna Porażka, Zenon J. Kokot, Edmund Grześkowiak

**Affiliations:** 1 Department of Clinical Pharmacy and Biopharmacy, Poznań University of Medical Sciences, ul. Św. Marii Magdaleny 14, 61-861 Poznań, Poland; 2 Polpharma Biologics, ul. Trzy Lipy 3, 80-172 Gdańsk, Poland; 3 Department of Inorganic and Analytical Chemistry, Poznań University of Medical Sciences, ul. Grunwaldzka 6, 60-780 Poznań, Poland; 4 Department of Animal Science, Iowa State University, 239E Kildee Hall, Ames, IA 50011 USA; 5 Hy-Line International, 2583 240th Street, Dallas Center, IA 50063 USA

**Keywords:** Lapatinib, Paracetamol, Paracetamol glucuronide and paracetamol sulphate pharmacokinetics, Drug-drug interaction

## Abstract

Lapatinib is a tyrosine kinase inhibitor used for the treatment of breast cancer. Paracetamol is an analgesic commonly applied to patients with mild or moderate pain and fever. Cancer patients are polymedicated, which involves high risk of drug interactions during therapy. The aim of the study was to assess the interaction between lapatinib and paracetamol in rats. The rats were divided into three groups of eight animals in each. One group received lapatinib + paracetamol (I_L + PA_), another group received lapatinib (II_L_), whereas the last group received paracetamol (III_PA_). A single dose of lapatinib (100 mg/kg b.w.) and paracetamol (100 mg/kg b.w.) was administered orally. Plasma concentrations of lapatinib, paracetamol and its metabolites – glucuronide and sulphate, were measured with the validated HPLC-MS/MS method and HPLC-UV method, respectively. The pharmacokinetic parameters of both drugs were calculated using non-compartmental methods. The co-administration of lapatinib and paracetamol increased the area under the plasma concentration-time curve (AUC) and the maximum concentration (C_max_) of lapatinib by 239.6% (*p* = 0.0030) and 184% (*p* = 0.0011), respectively. Lapatinib decreased the paracetamol AUC_0-∞_ by 48.8% and C_max_ by 55.7%. In the I_L + PA_ group the C_max_ of paracetamol glucuronide was reduced, whereas the C_max_ of paracetamol sulphate was higher than in the III_PA_ group. Paracetamol significantly affected the enhanced plasma exposure of lapatinib. Additionally, lapatinib reduced the concentrations of paracetamol. The co-administration of lapatinib decreased the paracetamol glucuronidation but increased the sulphation. The findings of this study may be of clinical relevance to patients requiring analgesic therapy.

## Introduction

Pain is a frequent symptom associated with cancer. Therefore, analgesic drugs are often administered to oncological patients [1]. Paracetamol effectively relieves pain due to selective inhibition of cyclooxygenase-2 (COX-2) and −3 (COX-3) in the central nervous system by interfering with descending serotoninergic pathways and, to some extent, by blocking the activity of pain mediators (bradykinine, substance P). Therapeutic doses are safe and tolerated well, but excessive intake of the drug may cause hepatotoxicity. Paracetamol is essentially metabolised in the liver. It is chiefly transformed by glucuronidation (40–60%) and sulphation (20–46%). In consequence, pharmacologically inactive metabolites are formed, while less than 10% is oxidised to a toxic metabolite, i.e. N-acetyl-p-benzoquinone imine (NAPQI) [2]. Many authors proved that paracetamol is a substrate of ABC transmembrane transporter – P-glycoprotein (P-gp). Many studies also revealed that paracetamol could modulate the P-gp activity. However, it is still unclear whether it is a potent inhibitor or inducer of this protein, and what factors may influence the direction of P-gp modulation by paracetamol.

Lapatinib is a small-molecule tyrosine kinase inhibitor (TKI), targeted at human epidermal growth factor receptor type 2 (HER2, ErbB2). It is used in combination with capecitabine, trastuzumab and aromatase inhibitors to treat breast cancer in HER2-positive women [3–5]. The metabolism of lapatinib is mediated mainly by CYP3A4 and CYP3A5, and to a minor extent – by CYP2C19 and CYP2C8 isoenzymes. Additionally, lapatinib was identified as a substrate of P-gp and BCRP (breast cancer resistance protein). It also inhibits the activity of P-gp, BCRP and OATP1B1. However, the clinical relevance of inhibition of the latter two transporters has not been elucidated. Some TKIs may inhibit UDP-glucuronyltransferase (UGT) and cause drug-drug interactions. In vitro and in vivo studies confirmed that nilotinib and erlotinib inhibited UGT1A, whereas gefitinib inhibited UGT1A1, 1A7, 1A9 and 2B7. There is not much data concerning the influence of lapatinib on the UGT activity. Zhan et al. [6] found that lapatinib inhibited glucuronidation of SN-38 (active metabolite of irinotecan) in human microsomes and recombinant UGT1A1 proteins. The quantitative prediction of drug-drug interaction risk indicates its clinical significance. Although Liu et al. [7] showed that lapatinib was a weak inhibitor of paracetamol glucuronidation, the *in vtiro* model of their study may underestimate inhibition of glucuronidation observed in vivo. Therefore, it is important to check the possible in vivo effect of lapatinib on paracetamol metabolism. The aim of this study was to investigate the influence of orally administered lapatinib on the pharmacokinetics of paracetamol and its glucuronidation and sulphation in rats. In addition, changes in the pharmacokinetic parameters of lapatinib after co-administration with paracetamol were analysed.

## Materials and methods

### Reagents

Lapatinib (CAS number 231277–92-9), paracetamol (CAS number 103–90-2), methanol, acetonitrile, formic acid, perchloric acid, theophyllinum, ammonium formate, and dimethyl sulfoxide were purchased from Sigma-Aldrich (Poznań, Poland). Erlotinib (CAS number 183321–74-6), paracetamol glucuronide and paracetamol sulphate were purchased from LGC Standards (Łomianki, Poland). Water used in the mobile phase was deionised, distilled and filtered through a Millipore system (Direct Q3, Millipore, USA) before use. Lapatinib (Tyverb®_,_ batch number Y68Y) was purchased from Novartis Polska Sp. z o.o., (Warsaw, Poland). Paracetamol (Pedicetamol, batch number K003) was purchased from Sequoia sp. z o.o., (Warsaw, Poland).

### Animals

The experimental protocol for this study was reviewed and approved by the Local Ethics Committee. All procedures were performed in accordance with the European Union regulations concerning the handling and use of laboratory animals. The study was based on the required minimum number of animals and observation time in order to obtain consistent data. Adult male Wistar rats (weight 420–505 g) were used in the study. The animals were maintained under standard breeding conditions with a 12/12 h light-dark cycle (lights on at 06.00, lights off at 18.00) at constant room temperature (23 ± 2 °C), relative humidity (55% ± 10%) and given ad libitum access to food and water. The animals were allowed to acclimatise for a week before the beginning of the experiments. The rats were divided into three groups. One group received lapatinib and paracetamol (I_L + PA_), another group received lapatinib (II_L_), whereas the last group received paracetamol (III_PA_). Lapatinib (100 mg/kg b.w. [8]) was dissolved in 1 mL DMSO (dimethyl sulfoxide) and administered directly into the animals’ stomachs using a gastric probe. In order to make sure that the animals received the entire dose of the drug, 1 mL of DMSO was then administered to rinse the probe. 80 μL of blood was collected from each rat by cutting off a piece of his tail. The blood samples were collected into heparinised test tubes at the following time points: 0, 15′, 30′, 1 h, 2 h, 3 h, 4 h, 6 h, 8 h, 12 h, 24 h. Paracetamol was administrated at a dose of 100 mg per kg of body weight [9] to the I_L + PA_ and III_PA_ groups. Blood samples (approximately 0.3 mL) for paracetamol analysis were collected before and 5, 15, 30, 60, 90, 120, 180, 240, 360 and 480 min after the drug administration. The blood samples were transferred into heparinised tubes and they were centrifuged at 2880 *g* for 10 min at 4 °C.

### HPLC-UV assay

The concentrations of paracetamol, paracetamol glucuronide and paracetamol sulphate were assayed using the HPLC method (high-performance liquid chromatography) with ultraviolet (UV) detection [10]. Separation was achieved by isocratic elution of the mobile phase, sodium sulphate 0.05 M pH 2.2 (adjusted with 85% orthophosphoric acid) – acetonitrile (93:7, *v*/v), at a flow rate of 1.5 mL/min through an ODS Hypersil® C18 column (150 mm × 4.6 mm, 5.0 μm particle size) (Thermo Electron Corporation®, Waltham, MA, USA). The total time of analysis for each run was 10 min. The column temperature was maintained at 25 °C, the UV detection wavelength was set at 261 nm, and the injection volume was 50 μL. The validation was performed according to the guidelines of the European Medicines Agency (EMA) concerning validation of bioanalytical methods. The lower limit of quantification (LLOQ) for paracetamol was 0.25 μg/mL, whereas for paracetamol glucuronide and paracetamol sulphate it was 1 μg/mL. Intra- and inter-day precision and accuracy of the LLOQ, low quality control (0.2, 2 and 2 μg/mL), medium quality control (30, 30 and 25 μg/mL), and high quality control (50, 50 and 40 μg/mL) were well within the acceptable limit of 11% coefficient of variation (CV%) and 10% of bias (% bias) for paracetamol, paracetamol glucuronide and paracetamol sulphate. The calibration curve for paracetamol was linear, within the range of 0.1–65 μg/mL (r = 0.995), for paracetamol glucuronide within the range of 1**–**60 μg/mL (r = 0.994) and for paracetamol sulphate within the range of 1–50 μg/mL (r = 0.998). 20 μL of the internal standard solution and 200 μL of 6% perchloric acid were added to 100 μL of the rat plasma. The samples were shaken for 30 s and then they were centrifuged for 10 min at 5000 *g*. About 100 μL of the resulting supernatants was transferred into glass inserts and a volume of 50 μL was injected onto HPLC column.

### HPLC-MS/MS assay

Lapatinib in the plasma samples was quantified using a high performance liquid chromatograph 1260 Infinity (Agilent Technologies, Santa Clara, CA, USA) combined with a triple quadrupole mass spectrometer 4000 QTRAP (Sciex, Framingham, MA, USA). Chromatographic separation was performed on a Kinetex C18 column (50 × 4.6 mm, 2.6 μm, Phenomenex, Torrance, CA, USA). The column temperature and injection volume were set at 35 °C and 10 μL, respectively. The mobile phase comprised 0.1% formic acid in water with 5 mM ammonium formate (eluent A) and acetonitrile with 10% phase A (eluent B). The flow rate was maintained at 700 μL/min and the gradient elution was as follows: 0–2 min, 95% A; 2–4 linear min from 95% to 5% A; 4–5 min, 5% A; 5–6 min linear from 5% to 95% A; 6–10 min, 95% A. The mass spectrometer operated in the multiple reaction monitoring mode. Two transitions for lapatinib and erlotinib (IS) were monitored: *m/z* 581.1 → 365.1 (quantifier transition) and 581.1 → 350.1 (qualifier transition) for lapatinib, and *m/z* 394.1 → 278.1 (quantifier transition) and 394.1 → 304.1 (qualifier transition) for IS.

An aliquot of the rat plasma (20 μL) was mixed with 50 μL of IS solution (100 ng/mL) and 930 μL of methanol and then vortexed for 30 s. As a result, a total dilution factor of 50 was obtained. After centrifugation at 10,000 *g* (10 min), the supernatant was transferred into amber glass HPLC vials. The validation was performed according to the guidelines of EMA concerning validation of bioanalytical methods. Different volumes of the lapatinib standard solution with 50 μL IS (c = 100 ng/L) were mixed in a total volume of 1.0 mL methanol to prepare calibration samples. The resulting correlation coefficient r > 0.995 ranged from 0.25 μg/L to 150 μg/L. The lower quantification limit was 0.25 μg/L. Quality control (QC) samples at four concentration levels (0.25; 0.5; 75; 125 μg/L) were obtained by spiking rat plasma samples with known quantities of lapatinib. The validation proved high precision (CV < 15%) and accuracy (%bias≤13%) of the methodology.

### Pharmacokinetic evaluation

The pharmacokinetic parameters were estimated with the non-compartmental method, using Phoenix® WinNonlin® 7.0 software (Certara L.P.). k_el_ – elimination rate constant; AUC_0-t_ – area under the plasma concentration-time curve from zero to the time of the last measurable concentration; AUC_0-∞_ – area under the plasma concentration-time curve from zero to infinity; t_0.5_ – elimination half-life; Cl/F – clearance; V_d_/F – volume of distribution; C_max_ – maximum plasma concentration; t_max_ – time necessary to reach the maximum concentration; MRT_0-t_ – mean residence time; AUMC_0-t_ – area under the first moment curve.

### Statistical analysis

The traits were tested for departure from normality using the Shapiro-Wilk test. The traits which did not show significant deviation from normality were subject to the heterogeneity of variance test, followed by pooled (heterogeneity of variance test *p*-value>0.05) or Satterthwaite (heterogeneity of the variance test *p*-value <0.05) t-tests to verify the significance of differences between the I_L + PA_ and II_L_ or I_L + PA_ and III_PA_. Differences between the I_L + PA_ and II_L_ or I_L + PA_ and III_PA_ in the characteristics which showed significant deviation from normality were tested with the Kruskal-Wallis test. The analysis was performed using capability t-test and npar1way procedures of SAS (SAS Institute Inc. 2002–2012. The SAS System for Windows version 9.4. Cary, NC, USA).

## Results

All the data were expressed as the mean value ± standard deviation (SD). The groups of rats did not differ significantly in the body mass. There was high intersubject variability between the groups, as reflected by the coefficient of variation (CV%).

## The influence of paracetamol on the pharmacokinetics of lapatinib

**Fig. 1:**
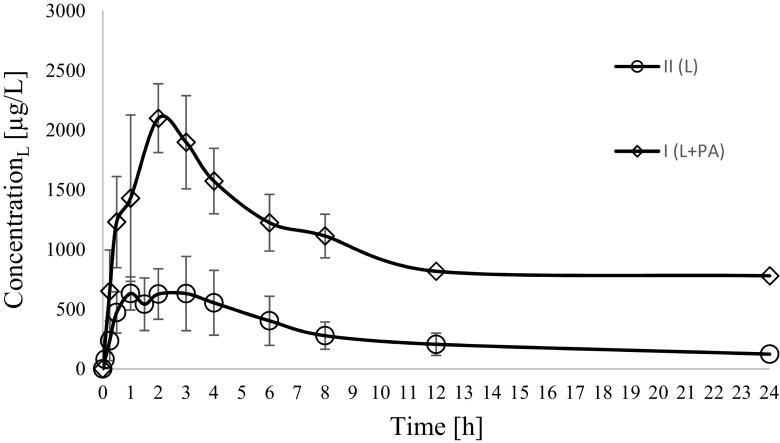
Lapatinib (L) plasma concentration–time profiles in rats receiving lapatinib + paracetamol (I_L + PA_) and lapatinib (II_L_)

Figure 1 shows the mean plasma concentration versus time profiles. Table 1 shows the associated pharmacokinetic parameters.

**Table 1:** Plasma pharmacokinetic parameters of lapatinib following a single *p.o.* dose of lapatinib of 100 mg/kg

Pharmacokinetic parameters^a^	I_L + PA_ M ± SD (CV%)	II_L_ M ± SD (CV%)	I_L + PA_ vs. II_L_ *p-value*
k_el_ (1/h)	0.07 ± 0.05 (79.2)	0.09 ± 0.03 (37.8)	0.4840^b^
AUC_0-t_ (mg × h/L)	20.67 ± 8.27 (40.0)	6.08 ± 3.93 (64.7)	0.0030^b^
AUC_0-∞_ (mg × h/L)	39.52 ± 23.96 (60.6)	8.04 ± 4.75 (59.0)	0.0065^d^
C_max_ (μg/L)	2235.50 ± 585.18 (26.2)	787.08 ± 213.40 (27.1)	0.0011^c^
t_max_ (h)	2.50 ± 0.84 (33.5)	2.42 ± 1.11 (46.1)	0.8864^b^
t_0.5_ (h)	16.23 ± 11.31 (69.7)	9.22 ± 3.93 (42.6)	0.2623^d^
Cl/F (L/h)	2.81 ± 1.20 (42.8)	11.46 ± 6.76 (59.0)	0.0065^d^
V_d_/F (L)	17.49 ± 2.55 (14.6)	57.22 ± 23.30 (40.7)	0.0039^d^
AUMC_0-t_ (mg × h^2^/L)	620.79 ± 1170.66 (188.6)	40.79 ± 37.98 (93.1)	0.0250^d^
MRT_0-t_ (h)	7.28 ± 3.15 (43.2)	5.77 ± 1.95 (33.8)	0.3418^b^

^a^AUC_0-t_. area under the plasma concentration-time curve from zero to the time of the last measurable concentration; C_max_. maximum plasma concentration; t_max_. Time to the first occurrence of C_max_; t_0.5_. half-life in elimination phase; Cl/F. clearance (Cl); V_d_/F. volume of distribution; AUMC_0-t_. area under the first moment curve from zero to the time of the last measurable concentration; MRT_0-t_. mean residence time;

^b^t-test; ^c^Satterthwaite test; ^d^Kruskal-Wallis test

The administration of paracetamol considerably increased lapatinib plasma concentrations. The mean *C*_max_ (*p* = 0.0011) and AUC_0 − ∞_ (*p* = 0.0065)in the lapatinib with paracetamol group were higher than in the lapatinib group. The increase in *C*_max_ by 284.0% as well as AUC_0 − ∞_ by 491.3% in the combination of lapatinib and paracetamol indicated that paracetamol significantly increased the bioavailability of lapatinib.

## The influence of lapatinib on the pharmacokinetics of paracetamol, paracetamol glucuronide and paracetamol sulphate

**Fig. 2:**
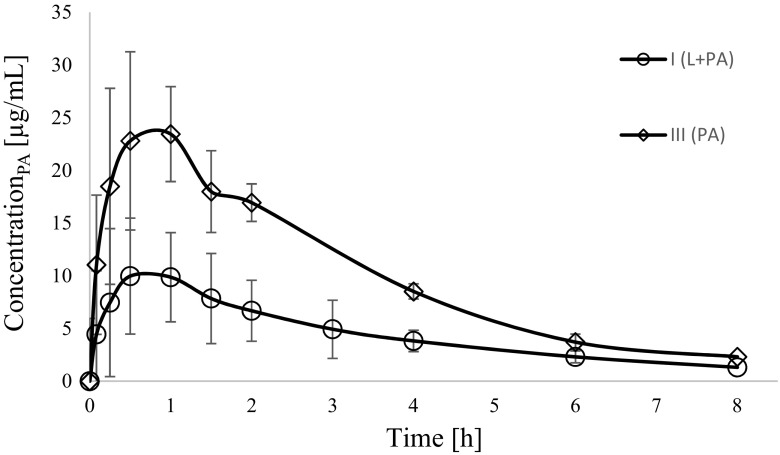
Paracetamol (PA) plasma concentration–time profiles in rats receiving paracetamol (III_PA_) and lapatinib + paracetamol (I_L + PA_)

**Fig. 3:**
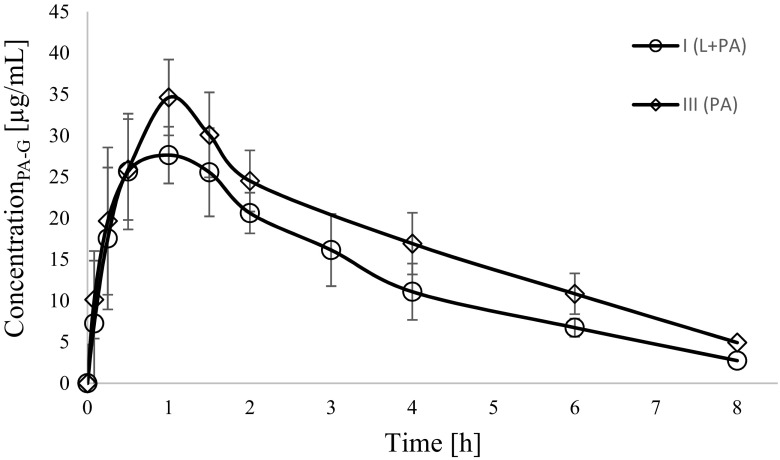
Paracetamol glucuronide (PA-G) concentration–time profiles in rats receiving paracetamol (III_PA_) and lapatinib + paracetamol (I_L + PA_)

**Fig. 4:**
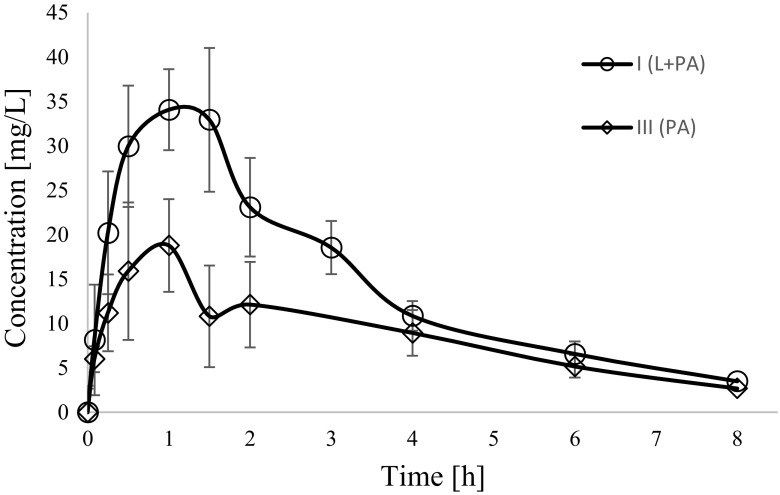
Paracetamol sulphate (PA-S) concentration–time profiles in rats receiving paracetamol (III_PA_) and lapatinib + paracetamol (I_L + PA_)

The mean arithmetic plasma concentrations of paracetamol, paracetamol glucuronide and paracetamol sulphate in the I_L + PA_ and III_PA_ groups are shown in Figs. 2, 3 and 4 respectively.

In comparison with the III_PA_ group the exposure to paracetamol in the I_L + PA_ group decreased, as evidenced by lower AUC_0-t_ (*p* < 0.0001), AUC_0-∞_ (*p =* 0.0004), C_max_ (*p* = 0.0030) and AUMC_0-t_ (*p* = 0.0023). There were also significant changes in the exposure of paracetamol metabolites between both groups. In the I_L + PA_ group the C_max_, AUC_0-t_ and AUC_0-∞_ of paracetamol glucuronide were reduced by 24%, 26%, 29%, respectively, whereas the C_max_, AUC_0-t_ and AUC_0-∞_ of paracetamol sulphate increased significantly by about 84%, 61%, 58%, respectively, as compared with the III_PA_ group (Table 2).

**Table 2:** Plasma pharmacokinetic parameters of paracetamol, paracetamol glucuronide and paracetamol sulphate following a single *p.o.* dose of paracetamol of 100 mg/kg

Pharmacokinetic parameters^a^	III_PA_ M ± SD (CV%)	I_L + PA_ M ± SD (CV%)	I_L + PA_ vs. III_PA_ *p-*value
	paracetamol		
k_el_ (1/h)	0.33 ± 0.10 (30.2)	0.24 ± 0.13 (53.2)	0.1259^b^
AUC_0-∞_ (μg × h/mL)	88.62 ± 8.96 (10.1)	45.41 ± 21.21 (46.7)	0.0004^c^
AUC_0-t_ (μg × h/mL)	80.46 ± 12.10 (15.0)	34.80 ± 20.07 (57.7)	<0.0001^b^
C_max_ (μg/mL)	24.70 ± 8.43 (34.1)	10.93 ± 6.88 (62.9)	0.0030^b^
t_0.5_ (h)	2.26 ± 0.70 (31.0)	5.30 ± 6.92 (130.7)	0.0929^d^
Cl/F (L/h)	0.57 ± 0.06 (9.9)	1.41 ± 0.87 (61.2)	0.0008^d^
V_d_/F (L)	1.98 ± 0.63 (31.6)	8.94 ± 8.04 (89.9)	0.0023^d^
AUMC_0-t_ (μg × h^2^/mL)	203.85 ± 18.15 (8.9)	97.11 ± 52.00 (53.6)	0.0023^d^
MRT_0-t_ (h)	2.57 ± 0.30 (11.5)	2.88 ± 0.27 (9.3)	0.0440^b^
	paracetamol glucuronide		
AUC_0-∞_ (μg × h/mL)	154.08 ± 35.58 (23.09)	109.94 ± 22.89 (20.8)	0.0105^b^
AUC_0-t_ (μg × h/mL)	136.24 ± 23.58 (17.31)	101.50 ± 22.02 (21.7)	0.0087^b^
C_max_ (μg/mL)	38.38 ± 3.75 (9.76)	29.01 ± 7.62 (26.3)	0.0075^b^
	paracetamol glucuronide/paracetamol^e^		
AUC_0-∞_	3.62 ± 1.72 (47.6)	3.36 ± 2.37 (70.6)	0.0274^c^
AUC_0-t_	1.75 ± 0.47 (27.1)	3.68 ± 1.67 (45.4)	0.0209^c^
C_max_	1.67 ± 0.41 (24.7)	3.58 ± 1.85 (51.5)	0.0356^c^
	paracetamol sulphate		
AUC_0-∞_ (μg × h/mL)	82.42 ± 32.71 (39.7)	130.23 ± 31.32 (24.1)	0.0098^b^
AUC_0-t_ (μg × h/mL)	71.92 ± 31.04 (43.2)	116.04 ± 27.02 (23.3)	0.0089^b^
C_max_ (μg/mL)	19.41 ± 7.05 (36.4)	35.15 ± 6.61 (18.8)	0.0004^b^
	paracetamol sulphate/paracetamol^f^		
AUC_0-∞_	1.05 ± 0.45 (43.1)	4.86 ± 2.74 (56.5)	0.0008^c^
AUC_0-t_	0.91 ± 0.43 (47.3)	4.27 ± 2.29 (53.5)	0.0008^c^
C_max_	0.88 ± 0.46 (52.7)	4.73 ± 3.02 (64.0)	0.0008^c^

^a^AUC_0-t_. area under the plasma concentration-time curve from zero to the time of the last measurable concentration; C_max_. maximum plasma concentration; t_max_. Time to the first occurrence of C_max_; t_0.5_. half-life in elimination phase; Cl. clearance (Cl); V_d_/kg. volume of distribution per kilogram; AUMC_0-t_. area under the first moment curve from zero to the time of the last measurable concentration; MRT_0-t_. mean residence time;

^b^t-test; ^c^Satterthwaite test; ^d^Kruskal-Wallis test; ^e^paracetamol glucuronide/paracetamol ratio; ^f^paracetamol sulphate/paracetamol ratio

## Discussion

Patients with metastatic breast cancer often suffer from pain at every stage of the disease. Paracetamol is one of the most common antipyretic and analgesic drugs. When paracetamol therapy follows the product characteristics, the risk of adverse effects is low. The most serious adverse reaction associated with paracetamol is hepatotoxic effect of one of its metabolites, i.e. N-acetyl-p-benzoquinone imine (NAPQI) [11]. Hepatotoxicity can be observed if there is insufficient amount of glutathione [12], e.g. when the drug has been overdosed or when the patient has consumed alcohol [13]. It is very likely that paracetamol and lapatinib will be applied simultaneously. Therefore, the possible interaction between the drugs may affect their pharmacokinetics.

The main goal of our study was to evaluate the influence of paracetamol on the pharmacokinetic parameters of lapatinib. We observed that the C_max_ of lapatinib and the exposure to the drug increased significantly in the I_L + PA_ group. These changes may be explained by increased inhibition of P-glycoprotein by paracetamol in the intestine. There were similar results in a study on rabbits receiving another TKI – erlotinib [14]. Studies on animals also revealed sex-dependent interactions with other drugs (paracetamol, ibuprofen, diclofenac, mefenamic acid) [15–17]. Paracetamol reduced male mice’s exposure to sunitinib by 29%, but did not affect the concentration of this TKI in female mice. However, it reduced both male and female mice’s exposure to the drug in their liver (by 15% and 9%, respectively), kidneys (by 15% and 20%) and brain (47% and 50%) [15]. Tan et al. [17] observed that the male mice which received paracetamol had 2.2 times higher liver sunitinib concentrations and 1.4 times greater kidney concentrations than the control group. These concentrations were significantly lower in the group of female mice. According to the authors, these changes may have been caused by sex-dependent differences in the activity of metabolising enzymes and proteins participating in the transport of drugs.

The aim of the second part of the study was to investigate the effect of lapatinib on the pharmacokinetics of paracetamol.

There were statistically significant differences in the C_max,_ AUC_0-t,_ AUC_0−∞_, AUMC_0-t,_ V_d_/F_,_ Cl/F and MRT_0-t_ values of paracetamol between the groups. The C_max,_ AUC_0-t_ and AUC_0−∞_ of paracetamol in the rats which received paracetamol and lapatinib were respectively about 60.5%, 56.8% and 48.8% lower than in the control group. The study also revealed a considerable increase in the V_d_/F and Cl/F values of paracetamol (351.5% and 147.4%, respectively) and a slight increase (12.1%) in the MRT_0-t_ of the drug.

There were also changes in the PK of paracetamol metabolites. The C_max,_ AUC_0-t_ and AUC_0−∞_ values of paracetamol glucoronide decreased by about 24.4%, 25.5% and 28.6% in the group of animals receiving both drugs, respectively. On the contrary, the C_max,_ AUC_0-t,_ and AUC_0−∞_ of paracetamol sulphate increased significantly (by 81.1%, 61.4%, 5.4%, respectively) in the animals from the I_L + PA_ group.

The abovementioned alterations in the PK of paracetamol and its main metabolites may have been caused by the inhibition of paracetamol biotransformation by lapatinib. Midgley et al. [18] observed a similar effect of lapatinib on the PK of irinotecan. They conducted a phase I clinical trial on the pharmacokinetics of lapatinib administered together with leucovorin, irinotecan and 5-fluorouracil and they observed that the AUC of the active irinotecan metabolite – SN-38 increased by about 40%. The authors suggested that the greater exposure to SN-38 may have been attributed to the inhibition of membrane transporters (OATP1B1, P-gp and BCRP) as well as metabolic enzymes - CYP3A4 and UGT1A1 by lapatinib [6]. Zhang et al. [6] confirmed that the inhibition of SN-38 glucuronidation was caused by considerable reduction of the UGT1A1 activity by lapatinib. The researchers also observed non-competitive UGT1A7 inhibition and mixed UGT1A4 inhibition [6].

Glucuronyl transferase UGT1A1 is one of the main enzymes responsible for glucuronidation of paracetamol [19, 20]. UGT1A1 isoform plays an important role in the metabolism of paracetamol, especially in case of high plasma concentration of the drug [20]. The inhibition of glucuronidation may redirect biotransformation of the drug to different pathways, such as increased conjugation with sulphuric acid, oxidation and formation of NAPQI. It is well known that lapatinib is a strong competitive inhibitor of glucuronyl transferase UGT1A1 [6]. However, to date there have been no available in vivo data concerning the effect of lapatinib on the paracetamol PK. Liu et al. [7] conducted an in vitro study and proved that other TKIs – sorafenib, dasatinib and imatinib, inhibited paracetamol glucuronidation in human liver microsomes. Similarly, axitinib, erlotinib, gefitinib, lapatinib, nilotinib and vandetanib exhibited a slight inhibitory effect against UGTs [7]. Importantly, the same authors emphasised the fact that particular caution should be taken when extrapolating in vitro studies to clinical situations. The study on the sunitinib and paracetamol interaction in rabbits proved that concomitant administration of the drugs (25 mg *p.o*., 35 mg/kg b.w. *i.v.,* respectively), contributed to lower exposure and faster clearance of paracetamol [21]. Furthermore, the same authors observed that another TKI – erlotinib, modified the metabolism of paracetamol, reducing its glucuronidation and intensifying its sulphation. Our study revealed changes in paracetamol metabolism which are consistent with other authors’ results and confirmed the inhibition of paracetamol glucuronidation by lapatinib on the animal model. Therefore, these observations should be verified on patients.

As was mentioned above, the alterations of metabolic pathways of paracetamol, especially the inhibition of glucuronidation, may lead to excessive synthesis of NAPQI, which is the most toxic metabolite [22] responsible for liver damage after the drug overdose. Ridruejo et al. [23] described the case of a 51-year-old female patient who was treated with imatinib administered at a dose of 400 mg/d due to chronic myelogenous leukaemia (CML). The overproduction of NAPQI, due to the inhibition of glucuronidation by imatinib, may have caused paracetamol-induced hepatotoxicity in the patient. Therefore, lapatinib may also exaggerate the hepatotoxicity of paracetamol. This hypothesis was supported by studies conducted by Moy et al. [24], who noted an isolated increase in the alanine aminotransferase (ALT) value, which was more than 3 times greater than the upper limit of normal values (ULN) in 3–5% of patients subjected to lapatinib therapy. The ULN was exceeded 5–20 times in 1.6% of the patients treated with lapatinib, whereas it caused hyperbilirubinemia and severe liver damage in 0.2% of the patients. Concomitant use of lapatinib and other cytotoxic drugs increases their hepatotoxicity due to the accumulation of the drugs in hepatocytes.

Manov et al. [25] and Pingli et al. [22] observed that the co-administration of P-gp inhibitors (verapamil, quinidine, ketoconazole and chrysin) increased the concentration and total exposure to paracetamol. This observation suggests that paracetamol is also a P-gp substrate. Thus, its toxicity may increase when it is administered with lapatinib, which is a strong inhibitor of this transporter.

## Conclusions

Due to the fact that the study proved higher concentration and exposure to lapatinib, there is also higher risk of lapatinib hepatotoxicity during a long-term combination therapy. It is especially important, because paracetamol is widely available as an over-the-counter drug. Patients may take paracetamol without consulting a physician, which may result in more frequent occurrence of adverse effects of both drugs. Patients should be carefully monitored to recognise symptoms of hepatotoxicity early. On the other hand, the reduced exposure to paracetamol and a considerable increase in clearance of the drug suggest that the analgesic activity decreases when paracetamol is administered together with lapatinib. The analgesic effect could be achieved by applying a higher dose of the drug or by reducing the dosage interval.
